# Effect of retained embryos on pregnancy outcomes of in vitro fertilization: a matched retrospective cohort study

**DOI:** 10.1186/s12884-022-05315-5

**Published:** 2023-01-04

**Authors:** Hui xia Zhang, Fei Li, Haixia Jin, Wen yan Song, Yingchun Su, Gang Li

**Affiliations:** 1 Centre for Reproductive Medicine, The First Affiliated Hospital of Zhengzhou University, Zhengzhou, China; 2 Henan Key Laboratory of Reproduction and Genetics, The First Affiliated Hospital of Zhengzhou University, Zhengzhou, China

**Keywords:** Retained embryo, Clinical pregnancy rate, Live birth rate, Embryo transfer, Operator

## Abstract

**Objectives:**

To explore the incidence of retained embryos (REs) in embryo transfer (ET) cycles and its effects on pregnancy outcomes in women undergoing in vitro fertilization (IVF).

**Methods:**

This was a matched retrospective cohort study involving 29,160 ET cycles conducted from March 2016 to February 2021, in which ET cycles without RE were matched to the RE group at a 2:1 ratio. Clinical pregnancy, implantation, miscarriage, and live birth rates were compared between the with-RE and without-RE groups.

**Results:**

Our study showed that the overall incidence of REs was 0.33% (95/29,160). There was a statistically significant difference in RE rate among the operators (*P* < 0.001), suggesting that the embryo retention rate may be affected by the individual operator. A total of 95 repeated ET cycles due to RE were included in the study group, and 190 ET cycles without RE were matched to the study group (1:2). There were no significant differences between the RE and matched groups in terms of implantation rate (35.6 vs. 38.0%; *P* = 0.608), clinical pregnancy rate (47.4 vs. 54.7%; *P* = 0.240), biochemical pregnancy rate (5.3 vs. 4.7%; *P* = 0.846), miscarriage rate (11.1 vs. 9.6%; *P* = 0.781), ectopic pregnancy rate (2.2 vs. 1.9%; *P* = 1.000) or live birth rate (41.1 vs. 48.9%; *P* = 0.208).

**Conclusions:**

The present findings demonstrated that immediate retransfer of REs did not significantly affect IVF outcomes, which may provide counselling information for patients when REs are identified and ET is reattempted. The incidence of REs was associated with the operator who expelled the embryos from the catheter. Attention to detail and frequent assessment of the operator’s technique may facilitate avoidance of embryo retention.

## Introduction

Embryo transfer (ET) is a crucial step in in vitro fertilization (IVF) [1]. Finding retained embryos (REs) in the transfer catheter after the first transfer attempt is an uncommon but clinically worrisome event, creating stress for patients and physicians. The reported RE rate varies from 0.7 to 7.5% [2, 3]. With the widespread use of ultrasound-guided ET, the incidence of REs has declined significantly over the past few decades [4, 5]. It has been suggested that contamination of the transfer catheter with mucus or blood, the number of embryos transferred, the embryonic stage and the technical difficulties at the time of ET were associated with the occurrence of retained embryos at transfer [2, 4–6].

Currently, immediate retransfer is universally adopted as a rescue method when embryos are retained in transfer catheter. However, there is a lack of consensus regarding whether retransferring REs has an impact on IVF outcomes, and patients and physicians still have uncertain expectations when a RE at transfer occurs. Several studies have reported that pregnancy outcomes were comparable when embryos were successfully transferred on the first attempt compared with those ETs that required multiple attempts due to embryo retention [2, 5, 7, 8]. However, Visser et al. [9] concluded that retention of embryos during transfer had an adverse impact on the pregnancy rate (20.3 vs. 3.0%). Recently, Xu et al. [10] found that retransfer of REs significantly decreased the implantation, clinical pregnancy and live birth rates. However, these studies had limited sample sizes and varied in study endpoint assessing IVF outcomes, and only three studies compared live birth rates between patients with and without RE [5, 7, 10]. Importantly, certain studies have not ruled out other factors that strongly influence IVF outcomes, such as maternal age, body mass index (BMI), type of cycle, embryo conditions and main causes of infertility. Therefore, the effects of REs on pregnancy outcomes warrant further investigation.

To better understand the potential implications of REs with regard to IVF outcomes, the present matched retrospective cohort study was conducted using a large sample of patients to investigate the association between embryo retention and pregnancy outcomes.

## Materials and methods

### Study design and cohort selection

This matched retrospective cohort study was based on the Clinical Reproductive Medicine Management System/Electronic Medical Record Cohort Database (CCRM/EMRCD) at our centre. We reviewed all IVF/intracytoplasmic sperm injection (ICSI)-ET cycles performed between March 2016 and February 2021. Cycles for preimplantation genetic testing, donor oocyte cycles and cycles without record of pregnancy outcome were excluded. Both fresh and frozen ET cycles were included in the study. All cycles were transferred with high quality embryo (grade I or grade II) [11] or blastocyst, morphological assessments of blastocyst were recorded based on the Gardner grading system [12]. In total, 95 ET cycles that had embryos retained in the transfer catheter and immediate repeat transfer were included in study group. The study was approved by the Ethics Committee of the First Affiliated Hospital of Zhengzhou University, and written informed consent was obtained from all participating subjects at the first consultation.

The matched group was selected according to the following criteria: female age (±1 year), female BMI (±2 kg/m^2^), type of cycle (fresh or frozen cycle), embryo condition (embryo stage, number, and quality), main cause of infertility (tubal factor, ovulatory dysfunction, diminished ovarian reserve, male factor, endometriosis, etc.), and type of protocol used for controlled ovarian hyperstimulation in fresh ET cycles or the endometrial preparation protocol in frozen ET cycles. We required exact matching for all the above criteria. To reduce the introduction of potential bias, researchers were blinded to reproductive outcomes during the matching process, and we sequentially selected each woman who met the inclusion criteria.

### Embryo transfer procedures

Each ET was carried out with a full bladder under transabdominal ultrasound guidance to allow for excellent visualization of the uterus and the transfer procedure. A Wallace catheter (Smith Medical International Ltd.) was used, this catheter system consists of an outer firm and an inner soft catheter. Patients were placed in the lithotomy position without any sedation or anaesthesia. After insertion of the speculum, the cervical mucus was removed using sterile cotton swabs with culture medium. An empty outer transfer catheter was passed through the external cervical os to the level of the internal cervical os. The tip of the catheter was placed approximately 1–1.5 cm from the uterine fundus under ultrasound visualization. The embryos were then loaded into an inner catheter with approximately 20 μL of medium that was flanked by small air bubbles. Air bubbles helped with ultrasound visualization. The inner catheter was then advanced until the desired intrauterine location was reached. The operator gently expelled the embryos from the catheter. The catheter was left in position for 1 min, after which it was withdrawn slightly and retrieved by the embryologist to check for retained embryos by microscopy. If any embryo was found to have been retained in the catheter, the retained embryos were immediately reloaded by the embryologist, and a second transfer was performed.

### Study variables and outcomes measures

The baseline demographic records for each patient were the female age, female BMI, infertility type, years of infertility, cause of infertility, transfer cycles, serum Anti-Müllerian hormone (AMH) and day-3 serum follicle-stimulating hormone (FSH). IVF cycle characteristics included the cycle type (fresh or frozen), stimulation protocol for fresh ET, endometrial preparation protocol for frozen ET, total gonadotropin (Gn) dose, endometrial thickness, number of oocytes retrieved, number of good-quality embryos and embryo stage.

The outcome measures in our study included biochemical and clinical pregnancy, implantation, ectopic pregnancy, miscarriage and live birth rate. The implantation rate was calculated as the number of gestational sacs per the number of transferred embryos. Clinical pregnancies were confirmed by ultrasonographic visualization of the gestational sac with a yolk sac, a foetal pole, and foetal heart pulsations. Biochemical pregnancy was defined as a pregnancy diagnosed only by the detection of beta hCG in serum per transfer. Ectopic pregnancies were diagnosed by ultrasound or laparoscopic visualization of an extrauterine gestational sac or by the absence of an intrauterine gestational sac and increasing hCG levels. Miscarriage was defined as spontaneous clinical pregnancy loss before 24 weeks of gestation. Live birth was defined as the birth of a live infant at ≥24 weeks of gestation. Birth weight and gestational age at delivery were recorded for all live-born infants.

A total of 8 operators performed ET during the study period, and they did not have previous experience in other institutions before entering the study. To determine whether the operator who expelled the embryos from the catheter was associated with the frequency of RE, we did comparisons among the RE rate of eight ET operators. The data on each ET operator included the number of procedures, date of entry, number of RE, number of procedures per year and rate of RE.

### Statistical analysis

SPSS 26.0 (IBM Co., Armonk, NY, USA) was used for statistical analyses. Continuous variables are presented as the mean ± standard deviation, and categorical variables are presented as frequencies (percentages). The differences in the pregnancy outcomes between groups were analyzed by the chi-square test or Fisher’s exact test. Comparisons of continuous parameters between groups were analysed by the independent samples t-test or the Mann–Whitney U test. The differences in the RE rate among ET operators were analyzed with the Fisher’s exact test. All tests were two-sided, and *P* < 0.05 was considered statistically significant.

## Results

A total of 29,160 ET cycles were performed by 8 operators from March 2016 to February 2021, including 13,638 fresh ET cycles and 15,522 frozen ET cycles. The overall incidence of RE during the above study period was 0.33% (95/29,160), and all retained embryos were immediately retransferred. In total, 5 of the 95 ET cycles required third transfer attempts, while the remaining 90 ETs were successful on the second attempt.

In all ETs, 190 ET cycles without RE matched to the RE group were selected (1:2). The baseline characteristics of the whole study population were presented in Table 1. The RE and matched groups were similar with regard to female age, BMI, infertility type, duration of infertility, cycle number, AMH, and day-3 serum FSH. The characteristics of these cycles are illustrated in Table 2. No significant difference was observed in type of cycle, stimulation protocol for fresh ET, endometrial preparation protocol for frozen ET, total dose of Gn, endometrial thickness, number of oocytes retrieved, number of good-quality embryos or embryo stage between the two groups. In the RE group, 72.6% were cleavage-stage ET (*n* = 65), 27.4% were blastocyst transfer (*n* = 25), 52.6% were fresh ET (*n* = 47) and 47.4% were frozen ET (*n* = 43).

**Table 1:** Patient demographic characteristics of two study group

Characteristics	RE group (***n*** = 95)	Matched group (***n*** = 190)	***P***-value
Age (years)	32.5 ± 5.3	32.4 ± 5.2	0.856
BMI (kg/m2)	23.7 ± 3.3	23.9 ± 3.2	0.793
Infertility type, n(%)			0.801
Primary	46(48.4%)	89(48.4%)	
Secondary	49(51.6%)	101(51.6%)	
Duration of infertility, n(%)			0.898
1–2	38(40.0%)	81(42.6%)	
3–5	40(42.1%)	78(41.1%)	
≧6	17(17.9%)	31(16.3%)	
Cause of infertility, n(%)			
Tubal factor	38(40.0%)	76(40.0%)	
Ovulatory dysfunction	15(15.8%)	30(15.8%)	
DOR or AMA	10(10.5%)	20(10.5%)	
Endometriosis	4(4.2%)	8(4.2%)	
Uterine	3(3.2%)	3(3.2%)	
Male factor	8(8.4%)	16(8.4%)	
Unknown factor	13(13.7%)	26(13.7%)	
Other	4(4.2%)	8(4.2%)	
No. of transfer cycles	1.6 ± 0.8	1.5 ± 0.7	0.612
Day 3 serum FSH (mIU/ml)	6.4 ± 2.3	6.9 ± 1.9	0.100
AMH (ng/ml)	3.7 ± 3.7	3.5 ± 3.6	0.627

Data are presented as mean ± SD for continuous variables and n(%) for categorical variables

*DOR* diminished ovarian reserve, *AMA* advanced maternal age, *AMH* anti-Mullerian hormone

**Table 2:** Cycle characteristics of the two study groups

Characteristics	RE group (***n*** = 95)	Matched group (***n*** = 190)	***P***-value
Fresh ET cycle, n(%)	50(52.6%)	100(52.6%)	
Frozen ET cycle, n(%)	45(47.4%)	90(47.4%)	
Stimulation protocol, n(%)			
Long agonist	44(88.0%)	88(88.0%)	
Antagonist	6(12.0%)	12(12.0%)	
Total dose of Gn (IU)	3049.4 ± 1408.2	2850.6 ± 897.5	0.280
Endometrium thickness (mm)	11.1 ± 3.2	10.8 ± 2.4	0.645
No. of oocytes retrieved	10.3 ± 6.1	11.3 ± 5.4	0.195
No. of good quality embryos	4.9 ± 3.2	4.8 ± 2.8	0.918
Protocol for frozen ET, n(%)			
Natural cycle	10(28.6%)	20(28.6%)	
Artificial cycle	35(77.8%)	70(77.8%)	
Cleavage-stage ET, n(%)	69(72.6%)	138(72.6%)	
Blastocyst transfer, n(%)	26(27.4%)	52(27.4%)	

Data are presented as mean ± SD for continuous variables and n(%) for categorical variables

*ET* embryo transfer, *Gn* gonadotropin

The pregnancy outcomes of the two groups after embryo transfer were displayed in Table 3. There were no significant differences between the RE group and matched group in terms of the implantation rate (35.6 vs. 38.0%, *P* = 0.608), clinical pregnancy rate (47.4 vs. 54.7%, *P* = 0.240), biochemical pregnancy rate (5.3 vs. 4.7%, *P* = 0.846), miscarriage rate (11.1 vs. 9.6%, *P* = 0.781), ectopic pregnancy rate (2.2 vs. 1.9%, *P* = 1.000), or live birth rate (41.1 vs. 48.9%, *P* = 0.208). In our study, the rate of pregnancy and live birth were lower among the REs cohort, but there was no significance. Furthermore, no significant difference was observed in gestational week at birth (37.8 ± 2.6 weeks vs. 38.6 ± 1.9 weeks, *P* = 0.115) or birth weight (3029 ± 570 g vs. 3175 ± 636 g, *P* = 0.213). No congenital birth defects were found in the RE group, although one congenital heart disease was observed in the matched group.

**Table 3:** Reproductive outcomes between RE group and matched group

Outcomes	RE group (***n*** = 95)	Matched group (***n*** = 190)	***P-*** value
Implantation rate	58/163 (35.6%)	123/324 (38.0%)	0.608
Clinical pregnancy rate	45/95 (47.4%)	104/190 (54.7%)	0.240
Biochemical pregnancy rate	5/95 (5.3%)	9/190 (4.7%)	0.846
Miscarriage rate	5/45 (11.1%)	10/104 (9.6%)	0.781
Ectopic pregnancy rate	1/45 (2.2%)	2 /104(1.9%)	1.000
Live birth rate	39/95 (41.1%)	93/190 (48.9%)	0.208
Gestational age (weeks)	37.8 ± 2.6	38.6 ± 1.9	0.115
Birth weight (g)	3029 ± 570	3175 ± 636	0.213

Data are presented as mean ± SD for continuous variables and (n/N)% for categorical variables

No significant difference was found between two groups

**Fig. 1:**
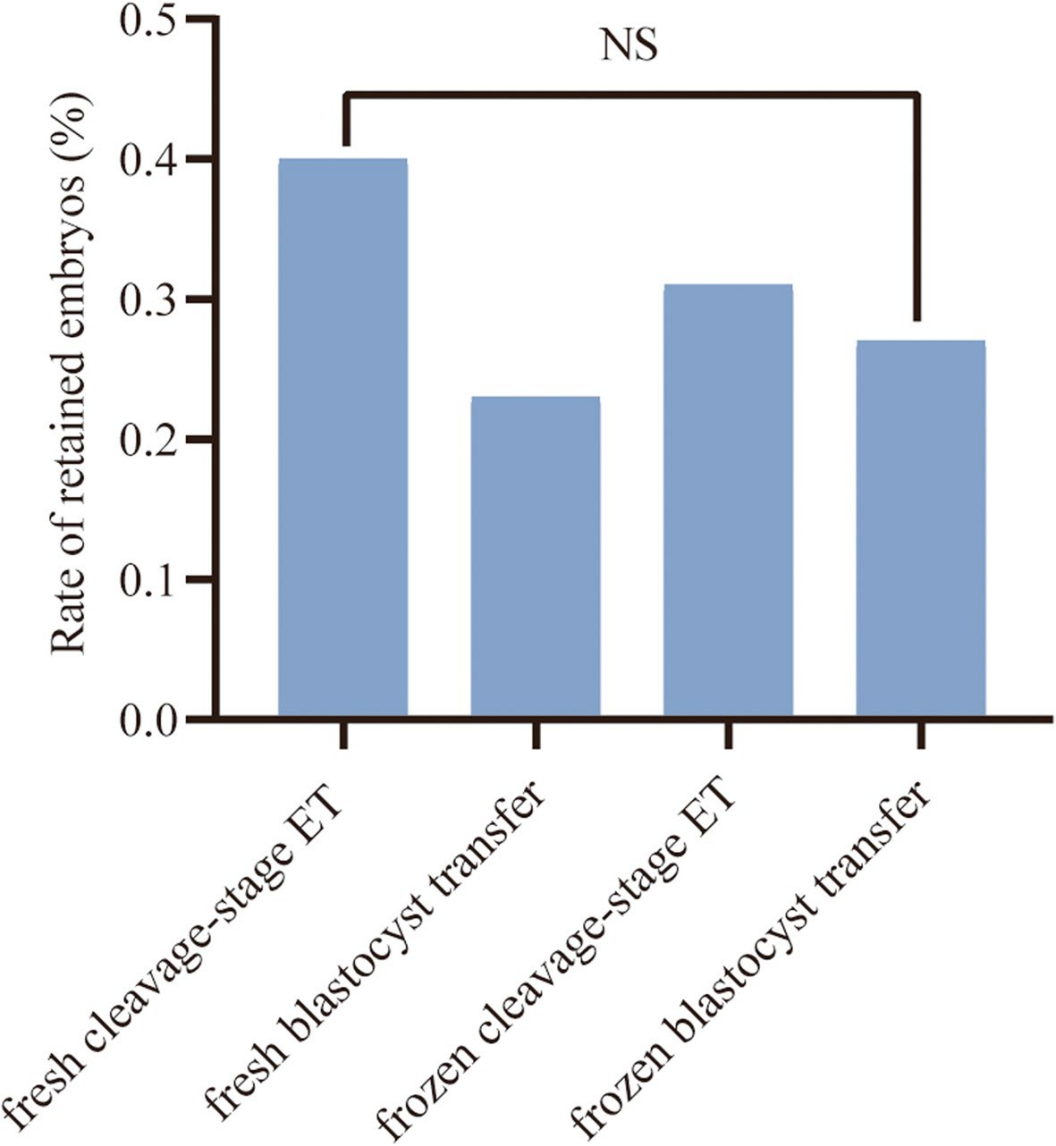
Comparisons of frequency of REs among the four ET strategies. NS indicates that no significant difference was detected among the four groups in terms of the RE rate. For ET cycles with REs, 44 (0.40%) were fresh cleavage-stage ET, 6 (0.23%) were fresh blastocyst transfer, 25 (0.31%) were frozen cleavage-stage ET and 20 (0.27%) were frozen blastocyst transfer

To detect possible risk factors that may contribute to the likelihood of RE, the RE rate in different ET processes was compared, including fresh cleavage-stage ET (*n* = 11,048), fresh blastocyst transfer (*n* = 2590), frozen cleavage-stage ET (*n* = 8071) and frozen blastocyst transfer (*n* = 7451). As shown in Fig. 1, there were no significant differences among the four groups in terms of RE rate (0.40 vs. 0.23 vs. 0.31 vs. 0.27%, *P* = 0.349).

**Fig. 2:**
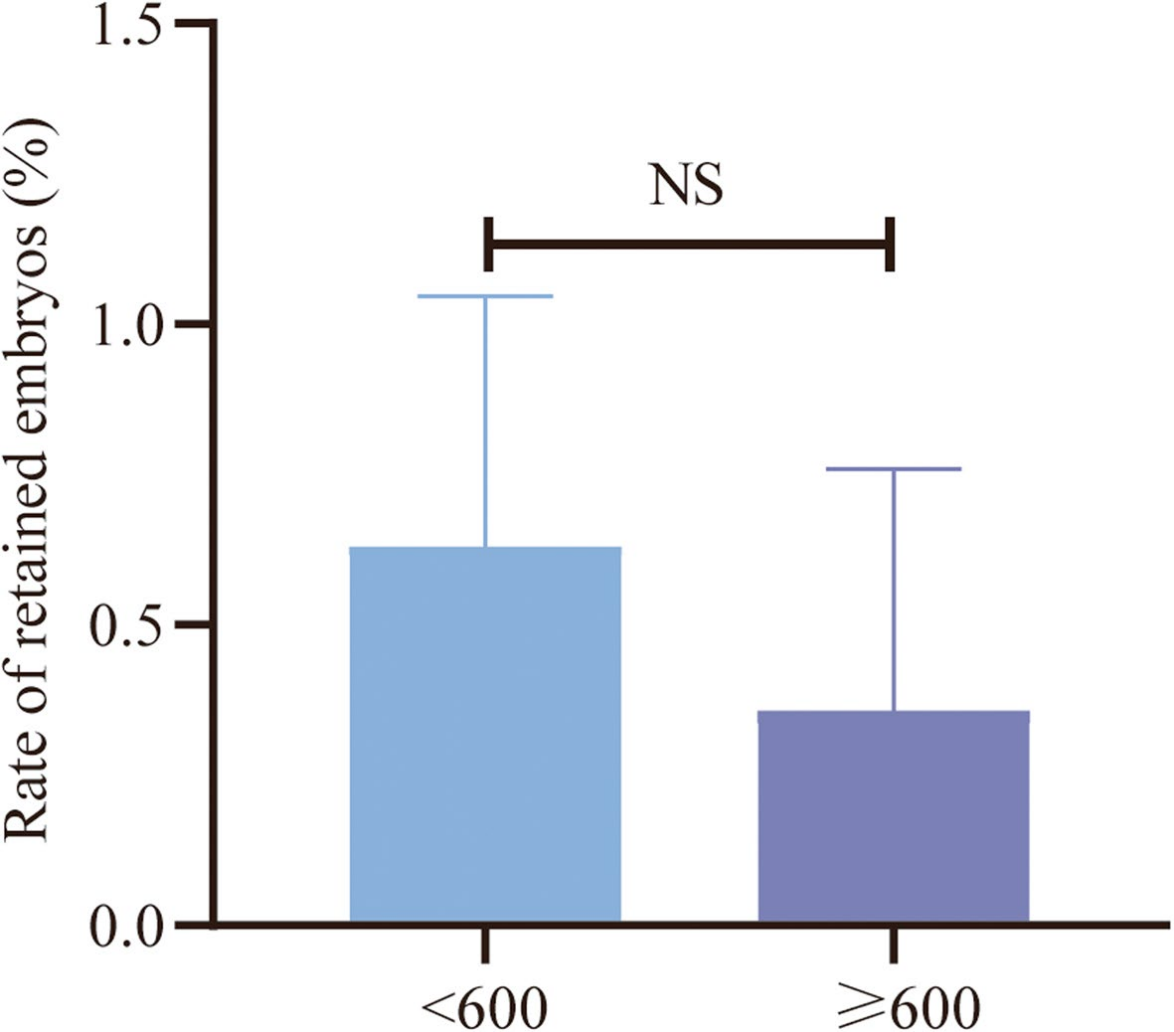
Comparisons of frequency of REs between the operators performing < 600 ETs per year and the operators performing≥600 ETs per year. NS indicates that no significant difference was detected between the two groups in terms of the RE rate

The present study retrospectively evaluated the individual operators’ impact on embryo retention rate. As shown in Table 4, operators who expelled the embryos from the transfer catheter were anonymized with a unique ID. In general, the 8 operators performed a mean of 1007 ± 770 ETs per year, and their overall RE rate was 0.49%, ranging from 0.10 to 1.21%. There was a statistically significant difference in RE rate among the operators (*P* < 0.001), suggesting that the embryo retention rate may be affected by the individual operator. In the present study, the operators’ experience was assessed in terms of numbers of procedures per year. The 600 procedures per year performed by the operators was chosen as the cut-off value in this study. According to this threshold, the operators were divided into two groups. As shown in Fig. 2, no significant difference was observed in rate of embryo retention between the two groups (*P* = 0.382), suggesting that the RE rate was not associated with the number of ETs performed per year.

**Table 4:** Association between the individual operator and retained embryos

Item	Number of procedures	Date of entry	Number of REs	Number of procedures/year	RE rate (%)	***P***-value
Operator ID						< 0.001
01	4330	3/2016	41	881	0.95	
02	9266	12/2016	11	2224	0.12	
03	8627	4/2017	9	2251	0.10	
04	2328	6/2017	6	635	0.26	
05	1370	6/2018	4	514	0.29	
06	1075	10/2018	13	461	1.21	
07	1087	12/2018	4	502	0.37	
08	1077	4/2019	7	587	0.65	
Total	29,160		95		0.49

## Discussion

It has long been debated whether retransferring REs could have a negative effect on pregnancy outcomes in women undergoing IVF. The present matched retrospective study suggested that IVF outcomes such as implantation, clinical pregnancy, miscarriage, live birth and ectopic pregnancy rates were not significantly affected by retransferring RE during ET. However, embryo retention remains an unwelcome event, and every effort should be made to achieve the lowest incidence of RE in ET.

The present study showed a relatively lower incidence of REs compared with that reported by other studies [8, 13]. Some studies found that having mucus in or on the transfer catheter was associated with a significantly increased rate of embryo retention, particularly in fresh ET cycles [10, 14]. Mucus may block the catheter opening or cause the embryo to adhere to the catheter when it is withdrawn [13]. When preparing for ET, attention must be paid to cleaning the vagina and external ostium with sterile warm water using sterile gauzes, and removing the cervical mucus using warmed sterile cotton swabs as needed. This may be a reason why the incidence of REs is relative lower in our centre. Furthermore, the current study demonstrated that transfer of blastocysts/cleavage-stage embryos and transfer of fresh/frozen embryos were not associated with the frequency of REs.

Data associated with impact of the individual operator performing ET on the RE rate are limited. A study carried out by Lee et al. [7] reported that, although the physician technique would be the most likely contributor to REs, the data did not show significant differences between physicians. In the current study, 8 operators who performed the ETs were involved in the analysis, which is a larger number than that included in previous studies [4, 10]. A statistically significant difference was found among the operators with regard to RE rate, suggesting that the operator who expelled the embryos from the catheter was associated with the occurrence of REs. In addition, the number of ETs performed per year was not found to be associated with the RE rate. The current findings agree with the literature, which reported that the operator’s ability to deposit embryos has been considered a crucial factor affecting the success of ET [15, 16]. Although ET is a standardized technique under ultrasound guidance, it can be affected by the operator’s manual ability, since they use their free hand to insert the preloaded soft catheter directly into the uterine cavity, expel the contents of the catheter, and then remove the catheter. Therefore, it was hypothesized that the skill of the operator performing ET, such as pushing force and controlled speed, could significantly affect the incidence of REs. To further decrease the RE rate, it may be necessary for operators to have periodic re-training or reassessment of their professional skills. Analysis and assessment of operator’s performance are performed regularly at our centre. Strict quality control management could help operators to test their expertise over time, correct poor performance and reduce the incidence of undesirable events. Furthermore, future research is necessary to further analyze the impact of individual operator, independent of all confounding factors including operator’s experience, on the incidence of REs.

Available published studies regarding the effects of REs on the IVF outcomes showed considerable differences in design, subjects and methods, yielding contradictory results. A previous study found that the embryo retention significantly reduced pregnancy rate (3 vs. 20.3%; *P* = 0.015) and recommended a 1-day delay before retransferring REs [9]. Notably, Visser et al. [9] included a small number of RE cases (*n* = 34) in their analysis, and only 1 case became pregnant. Several studies found that clinical pregnancy rates were lower in patients with RE compared with those in patients without retention of embryos, but not significantly different; however, in the study by Alvero et al., the immediate retransfer of retained embryos resulted in a significantly decreased implantation rate (17 vs. 31%; *P* = 0.03) [2–4, 7, 13]. Two of these studies only analyzed the effect of retained embryos at fresh ET on the pregnancy rates, while the other studies involved both fresh and frozen ET cycles [4, 13]. Three additional studies showed that there was no correlation between the retention of embryos during transfer and pregnancy rates [5, 8, 17]. Hyun et al. [5] included study subjects with and without blastocysts retained in the transfer catheter, which is different from other studies. Kaspa et al. [17] found that difficult transfers, transfers that required dilation of the cervix, or repeated transfers due to retained embryos had no adverse effect on pregnancy rates. It is well known that pregnancy rates after IVF are strongly affected by certain clinical parameters. However, the age and cycle characteristics were missing in 44.4% (4/9) of studies [3, 7, 9, 17]. Moreover, the number of transferred embryo was missing in 66.7% (6/9) of studies [3, 4, 7, 9, 13, 17], and six studies did not mention the embryo quality [3, 4, 7–9, 17]. Additionally, these studies did not consider the influence of age, cycle characteristics or condition of the transferred embryo in their analysis, which could also affect their results.

To minimize the impact of confounding variables potentially affecting the pregnancy outcomes of IVF, the current study used multiple matching criteria, such as female age, BMI, type of cycle, embryo condition, main causes of infertility and type of protocol used for fresh or frozen ET cycles. Xu et al. [10] used a study design similar to ours but obtained conflicting results. They found that patients with REs during ET had significantly lower rates of implantation (20.88 vs. 35.97%), clinical pregnancy (32.98 vs. 48.96%) and live birth (22.68 vs. 37.63%), and a higher rate of ectopic pregnancy (12.50 vs. 3.16%) than those without retention of embryos. We retrospectively analyzed the records of a considerable number of ET cycles to increase the accuracy of the findings. The current results may be explained by the following: Only a few retained embryos needed a third transfer, and almost all of them were successfully transferred after two attempts under the gentle operation of the physician. It is not suggested that the immediate retransfer of REs is necessarily related to the difficulty of the embryo transfer, which may require increased force or additional manoeuvres [18]. Previous studies have shown that difficult transfers significantly decrease the pregnancy rate, either by endometrial damage [19] or by the induction of uterine contractions that jeopardize correct embryo implantation [20]. The success of ET is related to embryo quality, endometrial receptivity and ET technique [21]. In our study, after strict matching for confounders, we did not find that REs inevitably had an adverse effect on pregnancy outcomes.

In the current study, there was one case of congenital birth defect in the matched group, and no significant difference was observed in the mean birth weight or mean gestational age at birth between the two groups. Thus, it seems that retransfer of REs does not negatively affect perinatal outcomes.

There are some strengths associated with the current study. First, our study reviewed a large cohort with 29,160 ET cycles, which likely reduced selection bias. Second, confounding variables potentially affecting the pregnancy outcomes of ET were matched between the two groups, thus making the outcomes independent of the baseline characteristics. Third, the operator who expelled the embryos from the catheter was found to be correlated with the incidence of REs, suggesting that the operator’s technique may be a significant risk factor for REs, which offers valuable information to assist in reducing the RE rate. Regarding limitations, it should be noted that our study did not evaluate the correlation between individual operator and pregnancy outcomes. We attempted to match the operator who performed ET as much as possible. However, since ET operators usually have a daily shift, they were not exactly matched between the two groups. It is possible that individual operators had an impact on the pregnancy rates, and this may represent a potential bias. Furthermore, the current study was a retrospective study conducted on a dataset from a single IVF centre. It is therefore possible that our analysis was affected by inclusion and selection biases.

In summary, our study demonstrated that the incidence of REs was associated with the operator who expelled the embryos from the catheter. Immediate retransfer of REs following the initial transfer attempt did not significantly affect pregnancy outcomes in patients undergoing IVF, which may provide counselling information for patients when REs are discovered and ET is reattempted. Regardless, retention of embryos in or on the transfer catheter is an undesirable event in clinical practice, and attention to detail and frequent assessment of operator’s technique may facilitate its avoidance. Due to the limited sample included in the present study, the effect of REs on pregnancy outcomes needs to be further investigated by expanding the sample size in future studies.

## Data Availability

The raw data supporting the conclusions of this article will be made available from the corresponding author on reasonable request.

## Abbreviations

REs: Retained embryos
ET: Embryo transfer
IVF: In vitro fertilization
BMI: Body mass index
ICSI: Intracytoplasmic sperm injection
AMH: Anti-Müllerian hormone
FSH: Follicle stimulating hormone
Gn: Gonadotropin
